# Age-Related Differences in Cortical Thickness Vary by Socioeconomic Status

**DOI:** 10.1371/journal.pone.0162511

**Published:** 2016-09-19

**Authors:** Luciane R. Piccolo, Emily C. Merz, Xiaofu He, Elizabeth R. Sowell, Kimberly G. Noble, Pediatric Imaging, Neurocognition, Genetics Study

**Affiliations:** 1 Department of Biobehavioral Sciences, Teachers College, Columbia University, New York, New York, United States of America; 2 Department of Epidemiology, Columbia University Medical Center, New York, New York, United States of America; 3 Department of Psychiatry, Columbia University, New York State Psychiatric Institute, New York, New York, United States of America; 4 Department of Pediatrics, Children’s Hospital Los Angeles, Los Angeles, California, United States of America; Institute of Psychology, Chinese Academy of Sciences, CHINA

## Abstract

Recent findings indicate robust associations between socioeconomic status (SES) and brain structure in children, raising questions about the ways in which SES may modify structural brain development. In general, cortical thickness and surface area develop in nonlinear patterns across childhood and adolescence, with developmental patterns varying to some degree by cortical region. Here, we examined whether age-related nonlinear changes in cortical thickness and surface area varied by SES, as indexed by family income and parental education. We hypothesized that SES disparities in age-related change may be particularly evident for language- and literacy-supporting cortical regions. Participants were 1148 typically-developing individuals between 3 and 20 years of age. Results indicated that SES factors moderate patterns of age-associated change in cortical thickness but not surface area. Specifically, at lower levels of SES, associations between age and cortical thickness were curvilinear, with relatively steep age-related decreases in cortical thickness earlier in childhood, and subsequent leveling off during adolescence. In contrast, at high levels of SES, associations between age and cortical thickness were linear, with consistent reductions across the age range studied. Notably, this interaction was prominent in the left fusiform gyrus, a region that is critical for reading development. In a similar pattern, SES factors significantly moderated linear age-related change in left superior temporal gyrus, such that higher SES was linked with steeper age-related decreases in cortical thickness in this region. These findings suggest that SES may moderate patterns of age-related cortical thinning, especially in language- and literacy-supporting cortical regions.

## Introduction

Experience-dependent plasticity has been found across many neural systems during childhood. At the cellular level, synaptic pruning is a hallmark of experience-dependent plasticity: repeated use strengthens synapses, while infrequent use leads to synaptic elimination [1–3]. Brain development may vary by socioeconomic status (SES) [4, 5], typically indexed by family income and/or parental education [6]. SES-related variability in children’s experiences has been associated with cognitive and social-emotional development throughout childhood and adolescence [7]. Some of the largest socioeconomic disparities have been found in the language domain [8]. Recent neuroimaging research has demonstrated that SES is associated with differences in children’s neural structure, especially in regions supporting language, memory and executive function [5, 8–12] raising questions about the ways in which family socioeconomic circumstance may modify developmental trajectories of brain structure.

In general, cortical structure develops nonlinearly and is influenced by both genetics and experience. Although most structural magnetic resonance imaging (MRI) studies have focused on cortical volume, this measure is a composite of cortical surface area (SA) and cortical thickness (CT), which are genetically and phenotypically independent [13–15]. SA and CT differ in their nonlinear developmental trajectories. SA expands through childhood and early adolescence and then decreases through middle adulthood [16–18]. In contrast, CT decreases rapidly in childhood and early adolescence, followed by a more gradual thinning, and ultimately plateauing in early- to mid-adulthood [15, 19–23]. These developmental changes in CT and SA are thought to relate to synaptic pruning and increases in white matter myelination [19, 21, 24–27]. Patterns of CT and SA development vary across cortical regions, with some regions exhibiting more nonlinear patterns of development than others [23, 28].

Studies of SES that have distinguished between SA and CT have reported that higher SES is associated with both greater SA [4] and greater CT [29, 30] in children and adolescents. For example, in a study of 3- to 20-year-olds, higher family income and parental education were significantly associated with greater SA, independent of age (including linear and quadratic terms), sex, genetic ancestry, and scanner [4]. Some research has also suggested that age-related changes in cortical and subcortical volume vary by SES [31, 32], particularly in language-supporting cortical regions [33]. For example, in a study of 5- to 17-year-old children, interactions between parental education and child age were found for volumes of the left superior temporal gyrus (STG) and left inferior frontal gyrus (IFG) [33]. Among higher SES children, relative regional volume increased with age, whereas for lower SES children, relative regional volume decreased with age (adjusted for total cortical volume) [33].

While previous work has suggested that linear age-related differences in SA and CT may be invariant across SES [4], little is known about how SES may modify *nonlinear* patterns of SA and CT development. This research question is an important one, because the shape of developmental trajectories may be a better indicator of differences in neurodevelopment than cortical differences at any single time point [34]. Differences in developmental trajectories have been found for children with psychiatric diagnoses [35–37], those with a history of prenatal alcohol exposure [34], and typically-developing children with different levels of general cognitive development [38]. Thus, investigating socioeconomic differences in patterns of age-related change across childhood and adolescence could lead to new insights about experience-related differences in structural brain development that underlie socioeconomic disparities in behavioral development.

As such, in this study of children and adolescents, we examined whether nonlinear developmental changes in CT and SA vary by SES. In separate models, we examined whether family income and parental education moderate the nonlinear association between age and mean CT and total SA. Family income and parental education were analyzed separately because they contribute distinctly to children’s development, at both the behavioral [39] and neural levels [40, 41]. We use vertex-based and region of interest (ROI) approaches to examine regions for which this interaction was significant. Based on prior studies [11, 41] we hypothesized that age by SES interactions would be prominent in the left hemisphere language cortex, including left fusiform gyrus, left IFG [42], and left STG [43].

## Method

### Participants

This study uses data from the multi-site Pediatric Imaging, Neurocognition, and Genetics (PING) study (http://ping.chd.ucsd.edu). As described in detail previously [4, 44] participants were recruited through a combination of web-based, word-of-mouth, and community advertising at nine university-based data collection sites in and around the cities of Los Angeles, San Diego, New Haven, Sacramento, Boston, Baltimore, Honolulu, and New York. Participants were excluded if they had a history of neurological, psychiatric, medical, or developmental disorders. All participants and their parents gave their informed written consent/assent to participate in all study procedures, including whole genome SNP genotype, demographic and developmental history questionnaires, and high-resolution brain MRI (see Table 1 for participant demographics). Each data collection site’s Office of Protection of Research Subjects and Institutional Review Board approved the original study. The current secondary data analyses were approved by the Teachers College, Columbia University Institutional Review Board (#16–103).

**Table 1:** Sample demographics (*N* = 1148).

	*M* (*SD*) or *n* (%)	Range
**Age in years**	12.05 (4.94)	3–20
**Sex**		
**Female**	554 (48%)	—
**Male**	594 (52%)	—
**Parental education in years**	15.03 (2.25)	6–18
**Family income in U.S. dollars**	97,617 (76,719)	4,500–325,000
**Genetic ancestry factor (GAF)**		
**African**	.13 (.26)	0–1
**American Indian**	.05 (.11)	0-.83
**Central Asian**	.03 (.13)	0–1
**East Asian**	.16 (.31)	0–1
**European**	.63 (.37)	0–1
**Oceanic**	.01 (.03)	0-.25

*Note*. GAF data show mean, standard deviation, and range across all subjects of the estimated proportion of genetic ancestry for each reference population. Descriptive statistics for demographics are provided for 1148 subjects, which is the maximum number of subjects used in analyses (as indicated in the Statistical Analyses section). U.S., United States.

### Socioeconomic status

As described in detail previously [4], parents were asked to report the level of educational attainment for all parents in the home. The average parental educational attainment was used in all analyses. Parents were also asked to report the total yearly family income. Data were not collected on the number of adults and children in the home, and thus we could not calculate income-to-needs ratios. Both education and income data were originally collected in bins, which were recoded as the means of the bins for analysis (see S1 Table). Family income was log-transformed for all analyses due to the typically observed positive skew. As expected, parent education and income were highly correlated (*r* = .526, *p* < 10^−6^). There were no SES differences in the sample by sex (parent education: *t*(1097) = 1.07, *p* = .28; family income: *t*(1097) = .19, *p* = .85). Parental education was associated with child age (*r* = −0.07, *p* < 0.05).

### Image acquisition and processing

Each site administered a standardized structural MRI protocol (see S2 Table for scanner models and parameters). Image acquisition and processing techniques have been described previously [44, 45]. Briefly, high-resolution structural MRI included a three-dimensional T1-weighted scan, a T2-weighted volume, and diffusion-weighted scans with multiple b values and 30 directions. In this paper, we focus on the T1-weighted images. All neuroimaging data passed a standardized quality control procedure. Image processing and analyses were performed using a modified FreeSurfer software suite (http://surfer.nmr.mgh.harvard.edu/) to obtain measures of cortical and subcortical volume and vertex-wise CT and SA [46]. Thirty-four cortical regions in each hemisphere were automatically parcellated by FreeSurfer using the Desikan-Killian Atlas [47], including the STG and fusiform gyrus. The IFG included the following Desikan-Killian parcellations: pars triangularis, pars orbitalis, and pars opercularis. CT of the IFG was computed by averaging CT for these parcellations, whereas SA of the IFG was computed by summing SA for these parcellations.

### Genetic collection and analysis

As described in detail previously [4], saliva samples were sent to Scripps Translational Research Institute (STRI) for analysis. Once extracted, genomic DNA was genotyped with Illumina Human660W-Quad BeadChip. Replication and quality control filters (that is, sample call rate >99, call rates >95%, minor allele frequency >5%) were performed [48]. To assess genetic ancestry and admixture proportions in the PING participants, a supervised clustering approach implemented in the ADMIXTURE software was used [49]. Using this approach, a GAF was developed for each participant, representing the proportion of ancestral descent for each of six major continental populations: African, Central Asian, East Asian, European, Native American and Oceanic. Implementation of ancestry and admixture proportions in the PING subjects is described in detail elsewhere [50]. A more complete description of the genetic ancestry of the PING sample is presented elsewhere [51].

### Statistical analyses

Children were nested within scanners within sites (nine total sites, 12 total scanners). Seven sites used one scanner, one site used two scanners, and one site used three scanners; thus, nesting within scanner and nesting within site were conflated. To account for nesting within scanner/site, multilevel modeling was conducted using SAS software (Version 9.3). To reduce multicollinearity and obtain standardized parameter estimates, all variables were standardized before running the models. As income was positively skewed, it was log-transformed, and the log of income was included in all of the models. For the parental education model, there were 1148 children with complete data on the relevant variables (i.e., age, sex, parental education, GAF, scanner/site, SA, and CT). For the family income model, there were 1138 children with complete data on the relevant variables (i.e., age, sex, family income, GAF, scanner/site, SA, and CT). For models that included both family income and parental education, there were 1099 children with complete data.

First, we examined SES x age^2^ interactions for mean CT and total SA using SAS software, as described above. Then, we used the PING data portal (http://pingstudy.ucsd.edu) to examine vertex-based regional specificity [52]. FDR correction was conducted at the .05 level. Based on prior studies [11, 33, 53], we also took an ROI approach and examined interactions in the left fusiform gyrus, left IFG, and left STG, which have been functionally and structurally associated with language and literacy [42]. For these three regions, we applied a Bonferroni correction and thus the p-level was set at .017 (.05/3). Because age-related patterns of change in cortical structure vary regionally [23, 28], we considered whether age was linearly or quadratically related to morphometry in each of these regions. When age^2^ was a significant predictor in the models, we examined the SES x age^2^ interaction term. When age^2^ was not significant, it was not included in the final model, and we examined the SES x age interaction instead.

To estimate effect size, we computed Cohen’s f^2^ [54], which is an effect size used to estimate the proportion of explained (vs. unexplained) variation uniquely accounted for by an independent variable over and above that accounted for by all other variables in the model [55]. Cohen’s f^2^ is interpreted by convention in terms of small (.02), medium (.15), or large (.35) effects [55].

## Results

### Family Income, Age, and Mean Cortical Thickness

**Fig 1:**
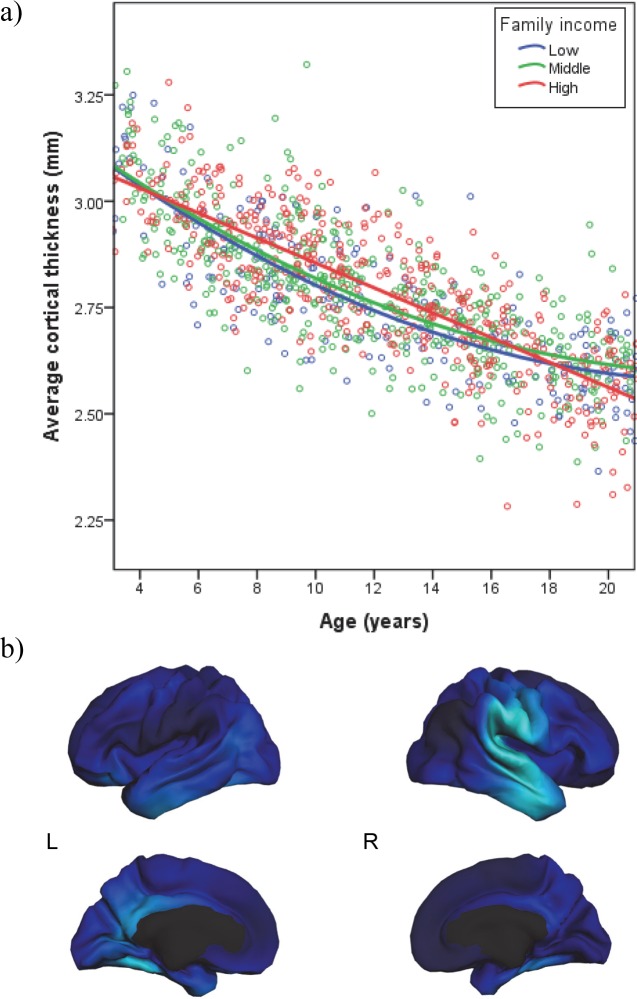
Family income significantly moderated non-linear age-related differences in mean cortical thickness (*N* = 1138). **(a)** Associations between age and average cortical thickness at low, middle, and high levels of family income. All analyses were performed using continuous variables for child age, family income, and cortical thickness, but are displayed in ecologically-valid family income groups ($4,500 - $25,000 in blue, $35,000 - $75,000 in green, and $125,000 - $325,000 in red). **(b)** The family income x age^2^ interaction for mean cortical thickness was mapped to visualize regional specificity. Although none of the associations survived FDR correction, regions significant at the .001 level are depicted here in light blue. These are regions where there is less curvilinearity in the association between age and mean cortical thickness with increasing family income.

Initial analyses revealed that models of mean CT were best fit using a quadratic function for age. We next assessed whether family income moderated the quadratic relationship between age and CT. Indeed, there was a significant family income x age^2^ interaction for mean CT, independent of family income, age, age^2^, family income x age, sex, GAF, and scanner/site (*β* = -.05, *p* = .0044, Cohen’s f^2^ = .02; see Table 2). Given that there were no significant GAF x family income interactions, these interactions were not included in the final model. As shown in Fig 1A, family income moderated the curvilinearity of the relationship of age to CT. Specifically, at low levels of family income, the relationship between and to CT is strongly curvilinear. As family income increased, the relationship between age and CT became increasingly linear.

**Table 2:** Family income by age^2^ interaction for average cortical thickness.

	*β*	*t*	*p*
**Sex**	.07	2.13	.0337
**GAF African**	-.06	-2.77	.0058
**GAF American Indian**	-.05	-2.59	.0098
**GAF East Asian**	-.09	-4.52	< .0001
**GAF Oceanic**	-.06	-3.37	.0008
**GAF Central Asian**	-.05	-2.79	.0053
**Family income**	.03	1.53	.1272
**Age**	-.78	-44.41	< .0001
**Age**^**2**^	.09	5.23	< .0001
**Family income x age**	-.01	-.79	.4319
**Family income x age**^**2**^	-.05	-2.85	.0044

*Note*. Multilevel modeling was used to control for the nesting of children within scanners/sites.

GAF, genetic ancestry factor.

### Post-hoc probing of significant interaction

Probing of this significant interaction (depicted in Fig 1A) was conducted using several methods [56, 57]. First, simple regression equations were computed showing the regression of CT on age and age^2^ at different levels of family income. The linear trend in the relationship of age to CT was similarly strong and negative across ecologically-valid levels of family income (low-income [$4,500-$25,000]; middle income [$35,000-$75,000], and high income [$125,000-$325,000]), *β* = -.77 to -.79, *p* < .001. Across income groups, CT tended to decrease with age across childhood and adolescence. However, the curvilinearity of this decrease varied by family income. Specifically, the regression of CT on age^2^ differed across levels of family income, *β* = .16 (*p* < .001) at low income, *β* = .11 (*p* < .001) at middle income, and *β* = .03 (*ns*) at high income.

Simple slopes were computed to estimate the linear association between age and CT at varying levels of both age and family income. For low family income, the steepness of the slope of CT on age decreased considerably with age, from -1.09 at 7 years, to -.77 at 12 years, and then -.44 at 17 years. Similarly, for middle family income, the steepness of the slope was -.99 at 7 years, -.78 at 12 years, and -.56 at 17 years. However, for high family income, the steepness of the slope of CT on age did not decrease much with age, from -.85 at 7 years, to -.79 at 12 years, to -.74 at 17 years. All of these slopes were significantly different from zero (*p* < .001). Taken together, these analyses provide statistical confirmation of the effects shown in Fig 1A.

**Fig 2:**
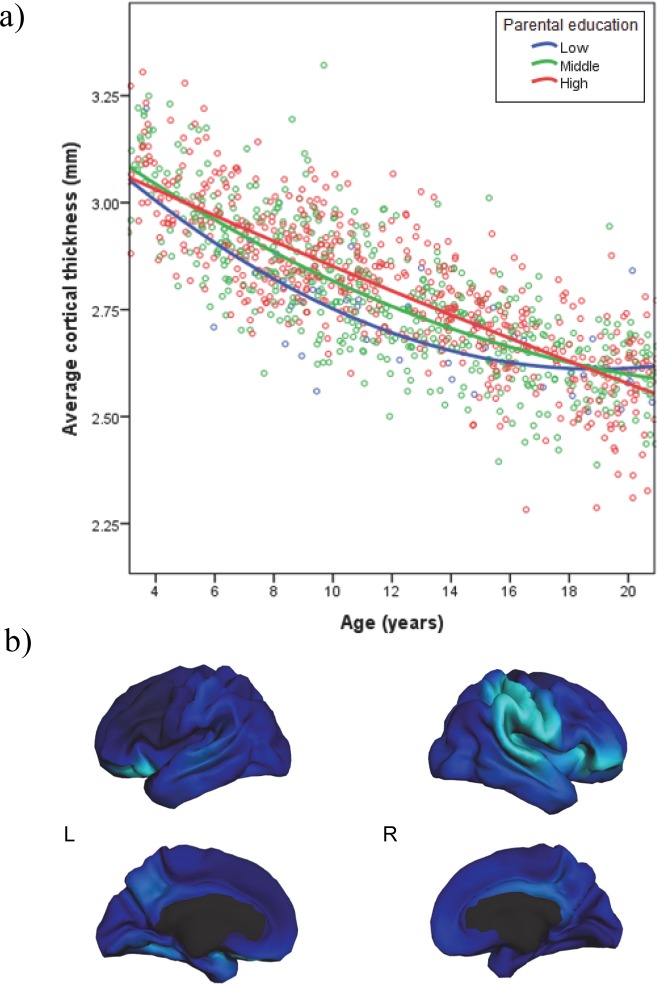
Parental education significantly moderated age^2^ for mean cortical thickness (*N* = 1148). **(a)** Associations between age and average cortical thickness at low, middle, and high levels of parental education. All analyses were performed using continuous variables for child age, parental education, and cortical thickness, but are displayed with parental education represented in ecologically-valid groups (less than a high school degree [6–11 years] in blue, high school or some college [12–14 years] in green, and 4-year college graduate or professional degree [16–18 years] in red). **(b)** The parental education x age^2^ interaction was mapped to visualize regional specificity. Although none of the associations survived FDR correction, regions significant at the .001 level are presented here in light blue.

Figs 1A and 2A show that before approximately age 18, children from more advantaged socioeconomic backgrounds tend to have thicker cortices, whereas after age 18, children from lower socioeconomic backgrounds tend to have thicker cortices. We therefore examined family income-related differences in mean CT in separate models for individuals who were younger and older than 18 years. Specifically, in each model, mean CT was regressed on family income as well as the covariates included in prior analyses (i.e., age, age^2^, sex, GAF, and scanner). In participants younger than 18 years (*n* = 926), individuals from lower-income families had lower mean CT compared to those from higher-income families (*β* = .008, *p* = .04). For those 18 years or older (*n* = 173), family income-related differences in mean CT were not significant, likely due to the reduced sample size in that group.

### Regional specificity

We next visualized the model to assess vertex-wise regional specificity of the family income x age^2^ interaction. When adjusting for all of the same covariates (i.e., family income, age, age^2^, family income x age, sex, GAF, and scanner), none of these associations survived FDR correction at the .05 level. However, based on prior work [11, 33], we hypothesized that SES would moderate age-related changes in CT in three cortical regions that support language and reading development: the left fusiform gyrus, left IFG, and left STG. As shown in Table 3, family income significantly moderated the age-related curvilinearity of cortical thinning in the left fusiform gyrus (*β* = -.08, *p* = .0009; Cohen’s f^2^ = .02). The pattern in the left fusiform gyrus showed the same pattern as was found for mean CT, with more curvilinearity of the relationship between age and CT at lower levels of family income.

**Table 3:** Family income x age^2^ interactions for cortical thickness of left hemisphere language regions.

	Left fusiform gyrus			Left IFG			Left STG		
	*Β*	*t*	*p*	*β*	*t*	*p*	*β*	*t*	*p*
**Sex**	.07	1.63	.1028	.06	1.45	.1476	-.03	-.55	.5850
**GAF African**	-.08	-2.81	.0050	.03	1.08	.2793	.01	.34	.7335
**GAF American Indian**	-.06	-2.28	.0231	-.03	-1.34	.1797	-.08	-2.87	.0042
**GAF East Asian**	-.10	-3.40	.0007	-.07	-2.56	.0106	-.11	-3.41	.0007
**GAF Oceanic**	-.07	-2.58	.0101	-.02	-.84	.4009	-.11	-3.81	.0001
**GAF Central Asian**	-.07	-3.20	.0014	-.05	-2.33	.0198	-.06	-2.43	.0152
**Family income**	.03	1.13	.2586	.05	1.88	.0607	.03	1.22	.2225
**Age**	-.58	-23.91	< .0001	-.65	-28.83	< .0001	-.47	-17.63	< .0001
**Age**^**2**^	.05	2.04	.0415	.08	3.65	.0003	—	—	—
**Family income x age**	-.004	-.19	.8515	-.03	-1.26	.2076	-.06	-2.45	.0145
**Family income x age**^**2**^	-.08	-3.34	.0009	-.03	-1.25	.2123	—	—	—

*Note*. IFG, inferior frontal gyrus; STG, superior temporal gyrus. Age^2^ was not significant for the left STG and thus was not included in these analyses.

Age^2^ was not a significant predictor of left STG CT, and therefore this variable was not included in the final model for this region (see Table 3). Family income significantly moderated linear age-related change in thickness of the left STG (*β* = -.06, *p* = .0145; Cohen’s f^2^ = .02), in a similar pattern to the left fusiform gyrus but without the curvilinearity. That is, at younger ages, higher family income was associated with a thicker cortex. Additionally, higher family income was linked with a more pronounced decline in CT with age. Thus, by mid- to late-adolescence, individuals from higher income families showed a thinner cortex in this region.

To inform future hypothesis generation, exploratory analyses across the entire cortex were conducted. Regions in which the family income x age^2^ interaction is significant when thresholded at the .001 level are presented in Fig 1B. In the left hemisphere, these included the fusiform gyrus, inferior temporal gyrus, isthmus cingulate, and posterior cingulate. In the right hemisphere, these included the fusiform gyrus, superior temporal gyrus, superamarginal gyrus, middle temporal, inferior temporal and postcentral gyrus.

### Parental Education, Age, and Mean Cortical Thickness

We next assessed whether parental education moderated the quadratic relationship between age and mean CT. As with family income, there was a significant parental education x age^2^ interaction for mean CT after adjusting for parental education, age, age^2^, sex, GAF, scanner/site, and parental education x age (*β* = -.05, *p* = .0028, Cohen’s f^2^ = .02; see Table 4). Given that there were no significant GAF x parental education interactions, these interactions were not included in the final model. The pattern of associations for this significant interaction closely resembled that of the family income x age^2^ interaction (Fig 2A). Specifically, at low levels of parental education, the association between age and CT was strongly curvilinear, but at higher levels of parental education the association becomes increasingly linear. Children of lower- and middle-educated parents show steep decreases in CT at younger ages, but then age-related cortical thinning slows by mid-adolescence. In contrast, children of highly educated parents show more gradual decreases in CT at younger ages, with continued evidence of cortical thinning through late adolescence.

**Table 4:** Parental education by age^2^ interaction for average cortical thickness.

	*β*	*t*	*p*
**Sex**	.06	1.74	.0824
**GAF African**	-.06	-2.99	.0029
**GAF American Indian**	-.06	-3.04	.0024
**GAF East Asian**	-.09	-4.60	< .0001
**GAF Oceanic**	-.07	-3.60	.0003
**GAF Central Asian**	-.05	-2.83	.0047
**Parental education**	.02	1.11	.2674
**Age**	-.77	-43.72	< .0001
**Age**^**2**^	.10	5.69	< .0001
**Parental education x age**	-.01	-.39	.6986
**Parental education x age**^**2**^	-.05	-3.00	.0028

*Note*. Multilevel modeling was used to control for the nesting of children within scanners/sites.

GAF, genetic ancestry factor.

#### Post-hoc probing of significant interaction

Probing of this significant interaction (depicted in Fig 2A) was conducted using the same methods as those described above [56]. First, simple regression equations indicated that the linear trend in the relationship of age to CT was similarly strong and negative across ecologically-valid levels of parental education (less than a high school degree [6–11 years of education], high school or some college [12–14 years of education] and 4-year college graduate or professional degree [16–18 years of education]), *β* = -.74 to -.78, *p* < .001. Thus, across levels of parental education, CT tends to decrease with age throughout childhood and adolescence. However, the curvilinearity of this decrease varied by parental education. Specifically, the regression of CT on age^2^ differed across levels of parental education, *β* = .25 (*p* < .001) at low educational attainment, *β* = .14 (*p* < .001) at middle educational attainment, and *β* = .06 (*p* < .05) at high educational attainment.

Second, simple slopes indicated that for low parental education, the steepness of the slope of CT on age decreased considerably with age, from -1.25 at 7 years, to -.74 at 12 years, and then -.23 at 17 years. Similarly, for middle parental education, the steepness of the slope was -1.05 at 7 years, -.76 at 12 years, and -.48 at 17 years. However, for high parental education, the steepness of the slope of CT on age did not decrease much with age, from -.89 at 7 years, to -.78 at 12 years, to -.67 at 17 years. All of these slopes were significantly different from zero (*p* < .05 to .001). Taken together, these analyses provide statistical confirmation of the appearance of Fig 2A. Additional post-hoc probing of the interaction revealed that there were no significant differences in CT across ecologically-valid parental education groups for individuals who were younger and older than 18 years.

#### Regional specificity

We then created maps to visualize the model to assess vertex-wise regional specificity of the parental education x age^2^ interaction. After adjusting for all the same covariates (i.e., parental education, age, age^2^, scanner, sex, GAF, and parental education x age), none of these associations survived FDR correction at the .05 level. We again examined whether this interaction was significant in our hypothesized ROIs. Indeed, as with family income, parental education significantly moderated quadratic age-related decreases in CT in the left fusiform gyrus (*β* = -.06, *p* = .0131; Cohen’s f^2^ = .02; see Table 5). The pattern in the left fusiform gyrus showed the same pattern as was found for mean CT, with more curvilinearity of the relationship of age to CT at lower levels of parental education.

**Table 5:** Parental education x age^2^ interactions for cortical thickness of left hemisphere language regions.

	Left fusiform gyrus			Left IFG			Left STG		
	*Β*	*t*	*p*	*β*	*t*	*p*	*β*	*t*	*p*
**Sex**	.06	1.32	.1882	.06	1.41	.1600	-.03	-.59	.5854
**GAF African**	-.07	-2.62	.0089	.01	.59	.5553	.01	.45	.6543
**GAF American Indian**	-.07	-2.44	.0150	-.05	-1.85	.0646	-.08	-2.80	.0052
**GAF East Asian**	-.11	-3.76	.0002	-.08	-2.97	.0030	-.10	-3.39	.0007
**GAF Oceanic**	-.07	-2.53	.0115	-.02	-.87	.3851	-.11	-3.73	.0002
**GAF Central Asian**	-.08	-3.33	.0009	-.05	-2.17	.0305	-.06	-2.42	.0156
**Parental education**	.04	1.47	.1422	.02	.81	.4209	.08	2.68	.0075
**Age**	-.57	-22.99	< .0001	-.65	-28.41	< .0001	-.46	-17.05	< .0001
**Age**^**2**^	.06	2.56	.0107	.08	3.45	.0006	—	—	—
**Parental education x age**	-.01	-.22	.8244	-.04	-1.69	.0910	-.07	-2.92	.0036
**Parental education x age**^**2**^	-.06	-2.48	.0131	-.03	-1.33	.1825	—	—	—

*Note*. IFG, inferior frontal gyrus; STG, superior temporal gyrus. Age^2^ was not significant for the left STG and thus was not included in these analyses.

Given that age^2^ was not a significant predictor of left STG CT, this variable was not included in the final model for this region. Parental education significantly moderated linear age-related change in the thickness of the left STG (*β* = -.07, *p* = .0036; Cohen’s f^2^ = .02; see Table 5), in a pattern that was similar to that of family income in this region. Specifically, higher parental education was associated with a thicker cortex in left STG at younger ages. Additionally, higher parental education was also linked with a steeper decline in CT with age, such that by late adolescence individuals from highly educated families had thinner cortices in this region.

Exploratory analyses across the entire cortex were conducted. Regions in which the parental education x age^2^ interaction is significant when thresholded at the .001 level are presented in Fig 2B. For the left hemisphere, these included the lateral orbital frontal cortex and fusiform gyrus. For the right hemisphere, these included the superior temporal gyrus, superamarginal gyrus, postcentral gyrus, lateral orbital frontal cortex, and inferior frontal gyrus.

### SES, Age, and Total Cortical Surface Area

**Fig 3:**
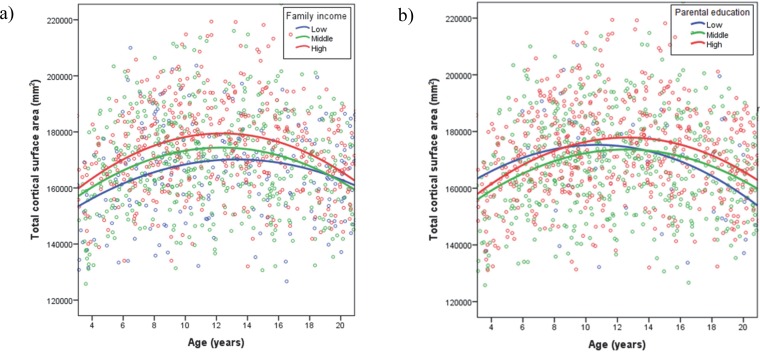
Associations between age and total cortical surface area at low, middle, and high levels of (a) family income and (b) parental education.

There were no significant family income x age^2^ or parental education x age^2^ interactions for total SA. As reported previously [4], there were significant main effects for family income, parental education, age, and age^2^. Fig 3A and 3B show the association between age and total SA for high, middle, and low levels of family income and parental education, respectively. As can be seen in these figures, the same curvilinear relationship between total SA and age is observed at each level of SES.

### SES, Age, and Hippocampal and Amygdala Volumes

Neither family income nor parental education moderated quadratic relations between age and either hippocampal or amygdala volumes. Previous work has found that SES moderates age-related decreases in hippocampal volume, but not amygdala volume, among older adults [32].

## Discussion

The present study investigated whether nonlinear age-related changes in CT and SA varied by SES, as indexed by family income and parental education. Results indicate that children from different SES backgrounds show different patterns of age-related change in CT but not SA. Consistent with previous research, in the sample overall, CT decreased with age. However, the association between age and CT was more curvilinear at lower levels of SES. Among lower SES children, CT declined steeply at young ages, followed by a more moderate decline, and then began to plateau in late adolescence. In comparison, among higher SES children, CT decreased at a steady rate throughout childhood and continued to decline at least through late adolescence, without plateauing.

In a vertex-based analysis, this interaction between age^2^ and SES did not show regional specificity at FDR levels of correction, likely because of the small effect size for the interaction. However, based on previous research [11, 41], analyses were conducted that focused on regions of interest in left hemisphere cortical regions supporting language and literacy. For one of these regions, the left fusiform gyrus, SES significantly moderated nonlinear CT development, in a pattern similar to that described above. In another of these regions, the left STG, there was not a significant quadratic component to age-related change in CT; rather, the thickness of this region declined linearly with age. Our results indicate that SES significantly moderated this age-related change in CT, such that higher SES was linked with a steeper decline in CT with age.

To our knowledge, this is the first study to examine the impact of SES on nonlinear age-related changes in cortical structure. Previous studies have demonstrated that higher SES is associated with greater CT in children. Specifically, in a study of 4- to 18-year-olds, higher parental education was associated with greater CT in prefrontal regions [29]. In a study of 13- to 15-year-olds, higher family income was associated with greater CT across all lobes of the brain [30]. Consistent with these findings, our results indicate that for the majority of childhood, higher SES does appear to be linked with greater CT, possibly due to a steeper rate of cortical thinning in children from lower-SES families early in childhood. However, this trajectory then changes in adolescence, when lower SES children begin to plateau and thus their CT stays at a higher level compared to higher SES children, for whom CT continues to decline.

SES may moderate patterns of cortical thinning in the left fusiform gyrus and the left superior temporal gyrus (STG), consistent with previous research showing SES differences in the structural development of language and literacy regions [41]. In fact, another study, focusing on children at-risk for reading impairment, reported that SES moderated the relationship between phonological language skills and reading-related brain activity in left fusiform and perisylvian regions. Specifically, among disadvantaged children, there was a strong association between language skill and activation in those regions during a reading task. However, as family SES increased, the association between language skill and activation in those areas was attenuated [11]. The left STG largely supports phonological processing, while the left fusiform gyrus supports visual word recognition [58–61]. Both of these are critical aspects of literacy development, which is an area of particular vulnerability for low-SES children. Indeed, some of the largest SES disparities are found in the language and literacy domain compared to other neurocognitive domains [62]. Thus, the current research may shed light on the underlying neurodevelopmental processes that may partially explain these differences.

These findings may reflect an abbreviated period of cortical thinning in lower SES environments, relative to a more prolonged period of cortical thinning in higher SES environments. It is possible that socioeconomic disadvantage is a proxy for experiences that result in a faster pace of cortical thinning (given the earlier plateau), whereas socioeconomic advantage allows for a longer window for this aspect of structural brain development to take place. It has been suggested that early adversity may narrow the sensitive period or time window for certain aspects of brain development that are malleable to environmental influences, thereby accelerating maturation [63, 64]. This phenomenon could potentially explain the pattern we report here, with children from more socioeconomically disadvantaged environments showing steeper age-related differences earlier in childhood, followed by a leveling off of thinning in adolescence, which is not observed in their higher SES peers.

There are some suggestions in the literature about the implications of individual differences in rates and patterns of cortical thinning over time. Some of this research has focused on the associations between rates of cortical thinning and general cognitive development (e.g., intelligence, IQ), which are complex and not yet well-understood. Greater cortical thinning has been related to increases in vocabulary [27] and improvements in cognitive and emotional control during adolescence [65–67], consistent with the direction of associations shown for adolescents in the present research. In addition, in a recent longitudinal study, greater early environmental stimulation in the home (at age 4) significantly predicted *reduced* CT in prefrontal and temporal regions in young adulthood, a finding which is consistent with results for older adolescents in the current study [68]. However, optimal rates of cortical thinning and levels of CT likely differ depending on variables such as the developmental period and brain region [19, 38, 69, 70]. Higher intelligence has also been associated with later timing of maturational changes [38]. It is possible, then, that the relatively prolonged thinning we find among children from higher socioeconomic families may in part account for widely-reported socioeconomic disparities in cognitive development and academic achievement.

Changes in CT may be due in part to synaptic pruning [25, 71, 72], which has been linked with the level and quality of stimulation in the environment [1, 3, 73]. High levels of stimulation strengthen synaptic connections, whereas low levels of stimulation may lead to excessive pruning. Further, the developmental timing of pruning is critical; pruning connections earlier in development that may be needed for future function would be counterproductive [24, 74, 75]. One possibility is that lower SES environments, which are often characterized by reduced cognitive and linguistic stimulation in and out of the home (e.g., [76]), may lead to greater pruning and thus reduced CT earlier in childhood. Changes in CT have also been linked with gliogenesis and increases in white matter myelination. Gliogenesis has been found to occur as a consequence of learning and experience [77] and is considered an important candidate mechanism for experience-related changes in gray matter morphology [78].

Both parental education and family income moderated age-related changes in CT. In previous studies, these two SES indices have been found to make distinct contributions to developmental outcomes [39]. Researchers have conjectured that family income may more directly reflect the physical resources available to the family in terms of enrolling the child in high-quality schools and providing enriching experiences. Parental education may more directly reflect parenting style and the quality of parent-child interactions [79]. Both of these dimensions of the environment have been linked with brain development [5], consistent with the present research.

In addition to the novelty of the research question, this study had a number of methodological strengths. The PING sample is one of the largest datasets available to date for neuroimaging research on brain development. In addition, our analyses were conservative in terms of ensuring parameter estimates were not inflated due to violating statistical assumptions, and analyses included a comprehensive set of covariates (e.g., sex, genetic ancestry, scanner/site). The inclusion of genetic ancestry, in particular, improves upon most prior studies of SES and brain development, because it more definitively rules out (genetic) race as a confounding factor.

This study also had limitations that should be kept in mind when interpreting the findings. The cross-sectional and non-experimental design of this study precludes any strong conclusions about causation. Analyzing cross-sectional data also has limitations in terms of drawing inferences about developmental processes [28, 80]. Longitudinal designs assessing within-subject change are necessary to supplement our findings for brain structure development. Because of the lack of longitudinal data, we were unable to address whether differences in rates and patterns of cortical thinning were associated with variability in cognitive outcomes. This study was also restricted to distal SES indices, specifically family income and parental education, in terms of moderating variables. It is important that future research examine the role of more proximal SES-related environmental factors, such as home environment and neighborhood quality, to further pinpoint the factors that may influence structural brain development.

## Conclusion

In sum, findings from this study indicated that age-related change in CT varies by SES, especially in regions supporting language and literacy. The curvilinearity of the association between age and CT decreased as SES increased, such that CT began to plateau during late adolescence for lower SES children but not higher SES children. Environmental differences associated with SES may influence aspects of structural brain development during childhood and adolescence. These results may contribute to our understanding of the neural mechanisms underlying socioeconomic disparities in cognitive development, and inform the design of effective prevention and intervention strategies which reduce these disparities.

## Supporting Information

S1 Appendix List of Group Authors. (PDF)

S1 Table Recoded Values for Parental Education and Family Income Bins. (PDF)

S2 Table Scanner Models and Parameters. (PDF)
