# Supplementary material for: Age-Related Differences in Cortical Thickness Vary by Socioeconomic Status
Source: PLoS One. 2016 Sep 19;11(9):e0162511. doi: 10.1371/journal.pone.0162511 (PMC5028041; doi:10.1371/journal.pone.0162511)
Supplement: S1 Appendix — (PDF) [file pone.0162511.s001.pdf]

AUTHORSHIP LIST FOR PING PUBLICATIONS

The Data and Publications Committee, in keeping with the publication policies adopted by the PING Executive Committee, here provide lists for standardized authorship. The list consists of two parts: Infrastructure Investigators and Site Investigators. Infrastructure Investigators are individuals responsible for leadership and infrastructure. Site Investigators are lead investigators at each recruiting site.

All papers using PING data, including methodological papers, should have an authorship list that consists of Infrastructure Investigators plus the FULL list.

Part A: Infrastructure

Coordinating Core:
Terry L. Jernigan, Ph.D., UC San Diego, Co-PI of PING, Core PI
Connor McCabe, B.S., UC San Diego

Assessment Core:
Linda Chang, M.D., U Hawaii, Co-PI of PING, Core PI
Natacha Akshoomoff, Ph.D., UC San Diego
Erik Newman, Ph.D., UC San Diego

MRI Post-processing Core:
Anders M. Dale, Ph.D., UC San Diego, Co-PI of PING, Core PI

MRI Acquisition Core:
Thomas Ernst, Ph.D., U Hawaii, Co-PI of PING, Core Co-PI
Anders M. Dale, Ph.D., UC San Diego, Core Co-PI
Peter Van Zijl, Ph.D., KKI
Joshua Kuperman, Ph.D., UC San Diego

Genetics Core:
Sarah Murray, Ph.D., Scripps Translational Science Institute, Co-PI of PING, Core PI
Cinnamon Bloss, Ph.D., Scripps Translational Science Institute
Nicholas J. Schork, Ph.D., Scripps Translational Science Institute

Informatics and Biostatistics:
Mark Appelbaum, Ph.D., UC San Diego
Anthony Gamst, Ph.D., UC San Diego
Wesley Thompson, Ph.D., UC San Diego
Hauke Bartsch, Ph.D., UC San Diego

Part B: Investigators by Data Collection Site

FULL PING Investigator Lists (3 author maximum per site):

University of California, San Diego:
Terry L. Jernigan, Ph.D.
Anders M. Dale, Ph.D.

Natacha Akshoomoff, Ph.D.

University of Hawaii:
Linda Chang, M.D.
Thomas Ernst, Ph.D.
Brian Keating, Ph.D.

University of California, Davis:
David Amaral, Ph.D.

University of California, Los Angeles:
Elizabeth Sowell, Ph.D.

Kennedy Krieger Institute, Johns Hopkins University:
Walter Kaufmann, M.D.
Peter Van Zijl, Ph.D.
Stewart Mostofsky, M.D.

Sackler Institute, Weill Cornell Medical College:
B.J. Casey, Ph.D.
Erika J. Ruberry, B.A.
Alisa Powers, B.A.

Massachusetts General Hospital, Harvard University:
Bruce Rosen, M.D., Ph.D.
Tal Kenet, Ph.D.

University of Massachusetts:
Jean Frazier, M.D.
David Kennedy, Ph.D.

Yale University:
Jeffrey Gruen, M.D.
