# Supplementary material for: Age-Related Differences in Cortical Thickness Vary by Socioeconomic Status
Source: PLoS One. 2016 Sep 19;11(9):e0162511. doi: 10.1371/journal.pone.0162511 (PMC5028041; doi:10.1371/journal.pone.0162511)
Supplement: S1 Table — (PDF) [file pone.0162511.s002.pdf]

S1 Table. Recoded values for parental education and family income bins

**Parental education bin**	**Recoded value**
Less than seven years of school	6
Seven to nine years of school	8
Ten to eleven years of school	10.5
High school graduate	12
Some college (1-3 years, AA, business schools)	14
Four-year college graduate (BA, BS, BM)	16
Professional degree (MA, MS, ME, MD, PhD, LLD, JD, etc.)	18
**Annual family income bin**	**Recoded value**
Less than $5,000	4,500
$5,000 - $9,999	7,500
$10,000 - $19,999	15,000
$20,000 - $29,999	25,000
$30,000 - $39,999	35,000
$40,000 - $49,999	45,000
$50,000 - $99,999	75,000
$100,000 - $149,999	125,000
$150,000 - $199,999	175,000
$200,000 - $249,999	225,000
$250,000 - $299,999	275,000
$300,000 and above	325,000
