# Supplementary material for: Age-Related Differences in Cortical Thickness Vary by Socioeconomic Status
Source: PLoS One. 2016 Sep 19;11(9):e0162511. doi: 10.1371/journal.pone.0162511 (PMC5028041; doi:10.1371/journal.pone.0162511)
Supplement: S2 Table — (PDF) [file pone.0162511.s003.pdf]

S2 Table. Scanner Models and Parameters

Site	Scanner Model	Scan Parameters
Massachusetts General Hospital	Siemens TrioTim	TR=2170ms, TE=4.33ms, TI= 1100ms, flip angle=7, matrix size=256x256, voxel size: 1x1x1.2, acquisition time: 8:06
Yale University		
University of California, Los Angeles		
University of California, Davis		
University of Hawaii		
Weill Cornell Medical College		
John Hopkins University<br>Children's Hospital, Los Angeles	Philips Achieva	TR=6.8ms, TE=3.1ms, TI= 845ms, flip angle=8, matrix size 256x240, voxel size=1x1x1.2, acquisition time: 9:19.7
University of California, San Diego	GE Signa<br>GE Discovery	TR=8.1ms, TE=3.5ms, TI=640 ms, flip angle=8, 256x192, voxel size: .94x1.2x1.2, acquisition time: 8:05

Note: Scanner Device Numbers, Models & Parameters for each site. All scanners are 3T strength. Parameters reported are for T1-weighted MPRage scans
